# Fluctuations between multiple EF-G-induced chimeric tRNA states during translocation on the ribosome

**DOI:** 10.1038/ncomms8442

**Published:** 2015-06-15

**Authors:** Sarah Adio, Tamara Senyushkina, Frank Peske, Niels Fischer, Wolfgang Wintermeyer, Marina V. Rodnina

**Affiliations:** 1 Department of Physical Biochemistry, Max Planck Institute for Biophysical Chemistry, Am Fassberg 11, Goettingen 37077, Germany; 2 3D Electron Cryomicroscopy Group, Max Planck Institute for Biophysical Chemistry, Goettingen 37077, Germany

## Abstract

The coupled translocation of transfer RNA and messenger RNA through the ribosome entails large-scale structural rearrangements, including step-wise movements of the tRNAs. Recent structural work has visualized intermediates of translocation induced by elongation factor G (EF-G) with tRNAs trapped in chimeric states with respect to 30S and 50S ribosomal subunits. The functional role of the chimeric states is not known. Here we follow the formation of translocation intermediates by single-molecule fluorescence resonance energy transfer. Using EF-G mutants, a non-hydrolysable GTP analogue, and fusidic acid, we interfere with either translocation or EF-G release from the ribosome and identify several rapidly interconverting chimeric tRNA states on the reaction pathway. EF-G engagement prevents backward transitions early in translocation and increases the fraction of ribosomes that rapidly fluctuate between hybrid, chimeric and posttranslocation states. Thus, the engagement of EF-G alters the energetics of translocation towards a flat energy landscape, thereby promoting forward tRNA movement.

## Abstract (web summary)

EF-G enhances the rate of tRNA–mRNA translocation on the ribosome. Here the authors use single-molecule FRET to follow tRNA translocation in real time, identifying new chimeric intermediates and suggesting how EF-G binding and GTP hydrolysis change the energetic landscape of translocation to accelerate forward tRNA movement.

## Graphical abstract

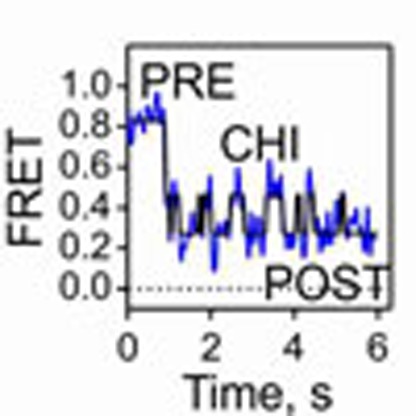

The elongation phase of protein synthesis entails the steps of mRNA decoding, peptide bond formation and translocation. During translocation, which in bacteria is promoted by elongation factor G (EF-G) at the cost of GTP hydrolysis, the tRNAs move from A to P and from P to E binding sites on the ribosome, carrying the mRNA along. There are several intermediate positions that the tRNAs can assume during the movement from the pretranslocation (PRE) to the posttranslocation (POST) state. The states known as classical (C) and hybrid (H) can interconvert spontaneously in the absence of EF-G^1^. In the C state, the deacylated tRNA and peptidyl-tRNA occupy the P/P and A/A positions, respectively, on the small 30S and large 50S subunit. In the H state, the 3′-ends of the tRNAs are shifted towards the 50S E and P positions, whereas the tRNA anticodons remain bound to the 30S P and A sites, respectively, and the ribosome adopts a conformation in which the subunits are rotated relative to one another^1^,^2^,^3^,^4^. In the absence of EF-G, C and H states are in dynamic equilibrium and populated to a comparable extent^5^,^6^,^7^,^8^. Their structures were solved by cryo-electron microscopy (cryo-EM) and crystallography^2^,^3^,^4^,^8^,^9^,^10^,^11^,^12^. There are further discrete tRNA sub-states on the trajectory from C to H, with the P-site tRNA moving gradually towards the P/E state, while the A-site tRNA remains in the C state^7^,^10^,^13^,^14^,^15^. The movements of the tRNAs are coupled to structural rearrangements of the ribosome. In addition to rotating relative to the 50S subunit, the 30S subunit undergoes internal motions (‘swiveling') of its head domain relative to the body^11^. On the 50S subunit, the dynamic L1 stalk fluctuates between open and closed conformations; these fluctuations are slow and coupled to the movement of the P/E-site tRNA^13^,^16^,^17^. Molecular dynamics simulations suggest that fluctuations of most other ribosomal elements are rapid (in the microseconds range) and spontaneous tRNA movement through the ribosome is restricted by the coupled dynamics of the tRNA_2_–mRNA module^18^.

EF-G is a five-domain GTPase that changes its conformation in response to GTP hydrolysis on the ribosome. Similar to all GTPases, EF-G has mobile switch 1 and 2 elements in its GTP-binding domain 1. The switch regions are disordered in unbound EF-G and become ordered when EF-G binds to the ribosome^19^,^20^,^21^. This transition causes a domain reorientation, such that the tip of domain 4 moves towards the 30S A site and intermediate structural states of the ribosome are stabilized. Mutations in EF-G that impair conformational changes in response to ribosome binding, GTP hydrolysis or the release of inorganic phosphate inhibit translocation^22^,^23^,^24^,^25^,^26^,^27^.

One cycle of unperturbed EF-G-mediated translocation is completed within milliseconds. After EF-G binding to the ribosome and GTP hydrolysis, coordinated rearrangements of the complex lead to mRNA unlocking on the 30S subunit and rapid, synchronous tRNA–mRNA movement on both 30S and 50S subunits into the POST state^23^,^28^. In the POST state, the peptidyl-tRNA occupies the P/P position and the ribosome assumes the classical, non-rotated conformation. At this stage, the peptidyl-tRNA has gained the ability to rapidly react with puromycin (Pmn), a mimic of the 3′-end of aminoacyl-tRNA used as a tool for monitoring 50S translocation. Binding of EF-G stabilizes the H state, presumably in a conformation where both tRNAs are in hybrid states, the subunits are rotated and the L1 stalk assumes the closed conformation^6^,^16^,^29^. EF-G also induces the formation of further sub-states, with tRNAs gradually moving with respect to the 30S and 50S subunits, designated, for example, ap/P, A/P2 or ap/ap states^23^,^30^,^31^. These intermediates with tRNAs in so-called chimeric states can be isolated by stalling the ribosome–tRNA–EF-G complex using (i) antibiotics, which inhibit translocation at different stages and by different mechanisms^32^; (ii) non-hydrolysable GTP analogues, which trap EF-G on the ribosome in a pre-hydrolysis state^19^,^20^,^21^; or (iii) EF-G mutants that inhibit translocation at particular steps^23^. However, little is known about the order of appearance of the various chimeric intermediates during unperturbed translocation, their dynamic properties, functional role, or how they contribute to the energy landscape of EF-G-catalysed translocation.

Here we analyse the positions of chimeric tRNA intermediates in the translocation landscape from the perspective of the A-site peptidyl-tRNA by monitoring single-molecule fluorescence resonance energy transfer (smFRET) using total internal reflection fluorescence (TIRF) microscopy. The presence of the A-site tRNA, or at least its anticodon–stem loop domain, is essential for translocation^33^, which underscores the importance of studying translocation intermediates of the A-site tRNA, beyond the known PRE (C and H) and POST states. To trap the intermediates, we employ various EF-G mutants, the non-hydrolysable GTP analogue GTPγS and the antibiotic fusidic acid (Fus), which disrupt the coupling between conformational rearrangements of EF-G and the ribosome in different, biochemically well-characterized ways (Supplementary Table 1). We (i) identify new intermediates of translocation from the perspective of the A-site peptidyl-tRNA over the whole trajectory towards the P-site; (ii) suggest the order of these intermediate tRNA states on the translocation reaction coordinate; (iii) show that these intermediate tRNA states can spontaneously and rapidly interconvert; and (iv) characterize how EF-G modulates these dynamics. These results, combined with information obtained by biochemical experiments and ensemble kinetics, suggest how the reaction energy landscape is changed by EF-G, thereby promoting forward translocation.

## Results

### Experimental setup for smFRET

**Figure 1:**
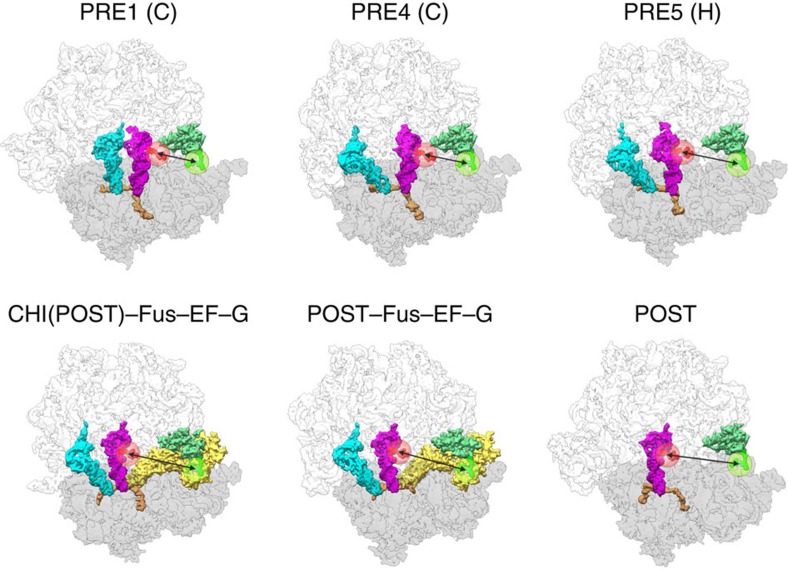
Expected distance changes between A-site tRNA and L11 on translocation. Distances between Cy5 attached to acp^3^U47 of fMetPhe-tRNA^Phe^ (raspberry) and Cy3 attached to Cys38 in L11 (green) in different PRE states (PRE1 (C), PRE4 (C) and PRE5 (H)) and POST states in the absence of EF-G (structures adapted from ref. 10), compared with the CHI and POST states with EF-G stabilized by Fus (CHI(POST)–Fus–EF-G^30^ and POST–Fus–EF-G^40^). The attached Cy3 and Cy5 dyes are shown in green and red, and the approximate average positions of the dyes are indicated by green and red circles, respectively. The 30S and 50S subunits are shown in grey and light grey, respectively. Double arrows indicate the expected increase of the distance between the two dyes on movement of the tRNA away from L11.

To monitor tRNA movements on the ribosome, we measured smFRET between fluorophores attached to peptidyl-tRNA and to ribosomal protein L11 in a TIRF microscope^34^,^35^. PRE complexes were assembled by mixing 70S initiation complexes containing fMet-tRNA^fMet^ and 5′-biotinylated mRNA (57 nucleotides) coding for fMetPhe with ternary complexes EF-Tu–GTP–Phe-tRNA^Phe^. After peptide bond formation, the PRE complexes contained tRNA^fMet^ in the P site and fMetPhe-tRNA^Phe^ in the A site. The activity of the ribosome complexes was verified by standard biochemical tests, including nitrocellulose filtration, dipeptide analysis and Pmn reaction^23^. Ribosome complexes were immobilized on the surface of a microscope slide by a biotin–streptavidin linkage at the 5′-end of the mRNA^5^. Fluorescence labels were specifically introduced into tRNA^Phe^ at the 3-(3-amino-3-carboxypropyl)uridine (acp^3^U) residue at position 47 (Cy5) and into protein L11 at the native Cys38 (Cy3; Fig. 1). Cy labels at those positions are well-characterized biochemically and were previously used to study translocation by smFRET^5^,^35^,^36^. Changes in Cy3–Cy5 FRET efficiencies report on movements of the elbow region of tRNA^Phe^ relative to the amino-terminal domain of L11. The *R*_0_ of the Cy3–Cy5 FRET pair, ∼60 Å (ref. 37), is optimally suited to monitor these movements, because the distance between the fluorophores changes from ∼50 Å in the PRE to 80 Å in the POST state^10^,^12^,^18^. Although L11 belongs to the L11-L10-L12 stalk, which is one of the mobile elements of the ribosome^38^, previous smFRET measurements suggested that only fluctuations between tRNA states were monitored (that is, C, H and POST states); additional fluctuations of L11 were not observed^34^. The fluorescence anisotropy of Cy3 attached to position 38 of L11 was close to the limiting anisotropy (Methods), indicating that the mobility of the dye and, with that, of the N-terminal domain of L11 was strongly restricted. In the presence of EF-G, the mobility of the N-terminal domain is probably further restricted by the interactions with EF-G^30^,^39^,^40^. Thus, FRET changes between Cy5 in the tRNA and Cy3 in L11 mainly reflect movements of the tRNA rather than of the N-terminal domain of L11.

### Structural dynamics of PRE and POST complexes

**Figure 2:**
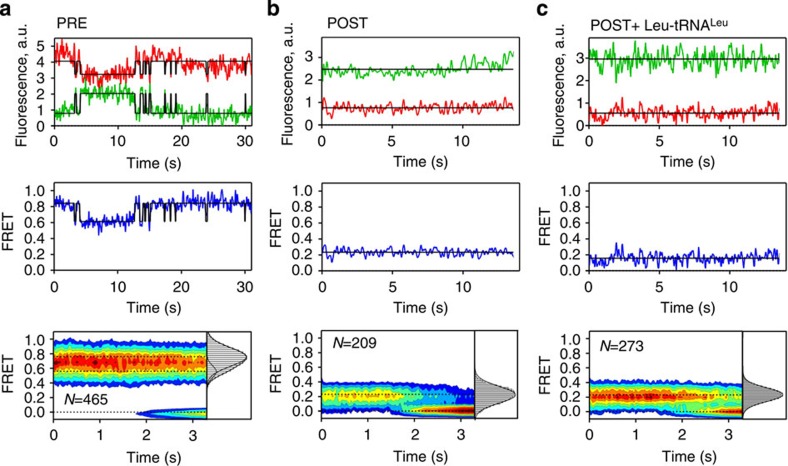
FRET between peptidyl-tRNA and protein L11 in PRE and POST complexes. (**a**) smFRET in the PRE complex. Representative examples of single-molecule fluorescence intensity trajectories for Cy3 (green) and Cy5 (red) (top panel), and the trajectory of smFRET over time (middle panel). Bottom panel: two-dimensional contour plot of smFRET in the PRE complex, revealing two major populations with FRET efficiencies of 0.76±0.12 (FRET 0.8 state) and 0.56±0.08 (FRET 0.6 state). The transition to FRET 0 represents photobleaching. (**b**) smFRET in the POST complex. Histogram and colour code as in **a**. The majority of the complexes are in a static state with FRET 0.24±0.11 (FRET 0.2 state). (**c**) Same as **b**, but in the presence of excess tRNA^Leu^ (2 μM) to occupy the E site. *V* values represent the number of smFRET trajectories used to construct contour plots and FRET distribution histograms (grey bars). a.u., arbitrary units.

Fluorophores were excited near the excitation maximum of Cy3 and emission intensities of Cy3 and Cy5 were monitored simultaneously near their respective emission maxima; from these traces, FRET trajectories over time were calculated (Methods; Fig. 2). For PRE complexes, the FRET efficiency was high, indicating a short distance between the fluorophores, as expected from the structures of the complexes (Fig. 1). Population distribution analysis revealed two sub-populations with FRET efficiencies of ∼0.8 and 0.6 (Fig. 2a). Half of the complexes fluctuated between FRET 0.8 and 0.6, whereas the others were static, that is, did not show any transition before photobleaching of the fluorophores (Supplementary Table 2). A distribution of fluctuating and non-fluctuating ribosome complexes has been observed previously^17^,^34^,^41^,^42^. At high Mg^2+^ concentration (15 mM) the FRET 0.8 state was predominant in both static and dynamic complexes, whereas at lower Mg^2+^ (7 mM) the two sub-populations were about equally represented (Supplementary Table 2). We observed the same number of states and FRET values also at a longer integration time (130 ms per frame instead of 33 ms, which were routinely used; Supplementary Fig. 1a). The existence of two sub-populations of the PRE complex and the dynamics of the transitions between them agree well with the transitions between C and H states observed in previous smFRET experiments^5^,^6^,^34^,^43^ or between the PRE1-4 and PRE5 sub-states identified by cryo-EM^10^. The POST complex, as obtained by the addition of a catalytic amount of EF-G together with GTP, was static (FRET 0.2) and showed no excursions to higher FRET states (Fig. 2b and Supplementary Fig. 1b). Binding of deacylated tRNA to the E site of the POST complex did not change the FRET efficiency (Fig. 2c). Thus, FRET efficiencies of 0.8 and 0.6 correspond to the C and H states of the PRE complex, respectively, whereas FRET 0.2 is characteristic for the POST state (with one or two tRNAs bound).

### Chimeric PRE states induced by EF-G

**Figure 3:**
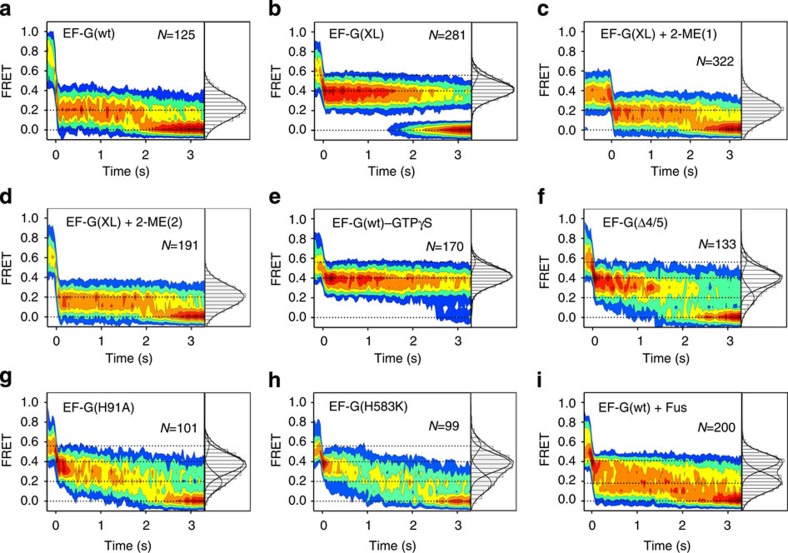
FRET changes during translocation. Data are displayed as contour plots after post synchronization. Fluorescence intensities and FRET trajectories are shown in Supplementary Fig. 2. Population distributions between the states and transition rates are given in Table 1. (**a**) After addition of EF-G(wt)–GTP. (**b**) EF-G(XL)–GTP. The FRET efficiency of the intermediate state was 0.40±0.09. (**c**) EF-G(XL) was activated by adding 2-mercaptoethanol (2-ME), while bound to the ribosome in the FRET 0.4 state. (**d**) EF-G(XL) was activated by 2-ME before binding to the ribosome. (**e**) EF-G(wt) with GTPγS. (**f**) EF-G(Δ4/5). (**g**) EF-G(H91A). (**h**) EF-G(H583K). (**i**) EF-G(wt) with Fus.

To determine trajectories of tRNA translocation, we injected EF-G–GTP into the flow chamber with immobilized PRE complex and monitored changes of the FRET signal in real time. For each PRE complex, we could distinguish a phase before EF-G recruitment, with FRET 0.8 or 0.6, and a subsequent translocation phase resulting in a decrease in FRET efficiency. To compare the translocation events, the trajectories were superimposed at the first transition from high FRET to FRET≤0.5, such that the onset of translocation could be back-tracked for each trace (‘post synchronization')^5^. With wild-type (wt) EF-G and GTP, translocation proceeded from the PRE states with FRET 0.8 or 0.6 to the POST state with FRET 0.2 within one frame (33 ms), without showing any discernible intermediates (Fig. 3a). This result is in agreement with the rapid kinetic studies, which suggested that translocation takes place rapidly and synchronously on both ribosomal subunits^23^,^28^. To isolate translocation intermediates, we first used an EF-G mutant, EF-G(XL), in which the mobility of domains 1 and 5 is restricted by a reversible disulfide cross-link between the two domains^24^. EF-G(XL) binds to ribosomes, hydrolyses GTP and releases inorganic phosphate as wt EF-G, whereas it is inhibited in subsequent steps of translocation. Binding of EF-G(XL) to the PRE complex revealed a stable intermediate state of translocation with FRET 0.4 (Fig. 3b and Table 1). At conditions where the disulfide cross-link was intact, translocation transitions to the final POST state were not observed. When the disulfide bridge was reduced by the addition of 2-mercaptoethanol, the FRET 0.4 state was readily converted to the FRET 0.2 (POST) state, suggesting that the FRET 0.4 state is an authentic translocation intermediate (Fig. 3c). When the disulfide bridge in EF-G(XL) was dissolved by 2-mercaptoethanol before binding to the ribosome, the transition was identical to that observed with wt EF-G, as expected (Fig. 3d). Although the FRET 0.8, 0.6 and 0.2 states—in the simplest model—can be attributed to the PRE (C), PRE (H) and POST states, respectively^7^,^10^,^34^, the FRET 0.4 state appears to be a novel intermediate that has not been observed previously, perhaps because different label positions and/or a lower frame rate was used^34^.

To search for other potential translocation intermediates, we used a non-hydrolysable analogue of GTP, GTPγS and several EF-G mutants known to stall translocation (Supplementary Table 1). Among the various non-hydrolysable GTP analogues, GTPγS structurally is closest to unmodified GTP and its hydrolysis is slow enough (0.005 s^−1^ at 37 °C) not to interfere with its use for translocation experiments. EF-G(wt) in the presence of GTPγS, which promotes slow translocation on both 30S and 50S subunits^44^, induced a long-lived FRET 0.4 intermediate with only very few transitions to the FRET 0.2 state (Fig. 3e and Table 1). The FRET 0.4 state was also induced by EF-G lacking domains 4 and 5 (Fig. 3f). In that case, three FRET states, 0.6, 0.4 and 0.2, were populated (Table 1). EF-G(Δ4/5) is not impaired in GTP hydrolysis on the ribosome, but causes extremely slow tRNA movement^22^,^23^,^25^. The estimated overall rate of translocation with either GTP(wt)–GTPγS or EF-G(Δ4/5)–GTP (≤0.07 s^−1^; Table 1) is in reasonable agreement with the rates estimated from ensemble kinetics: 0.1 s^−1^ at 20 °C for EF-G–caged GTP, another non-hydrolysable GTP analogue, which affects translocation in a similar way as GTPγS^44^,^45^, and 0.06 s^−1^ for EF-G(Δ4/5)^23^, respectively. EF-G mutants with amino acid replacements H91A in domain 1 (refs 22, 23) or H583K in domain 4 (ref. 25) transiently stall translocation at a stage where the 3′-end of peptidyl-tRNA occupies an intermediate position (INT) between the A- and P-sites on the 50S subunit. At the same time, translocation on the 30S subunit and the final movement of the 3′-end into the Pmn-reactive POST state in the 50S P site are slow, about 1 s^−1^ (Table 1 and refs 22, 23). With both EF-G mutants, the FRET 0.4 intermediate was predominant; however, transient fluctuations to FRET 0.6 and 0.2 were also observed (Fig. 3g,h). Finally, when translocation was carried out with wt EF-G in the presence of Fus, a mixture of interconverting FRET 0.4 and 0.2 states was found, but no excursions to FRET 0.6 were observed (Fig. 3i and Supplementary Fig. 2i). Thus, the transient state of peptidyl-tRNA with FRET 0.4 appears to be an authentic translocation intermediate between PRE (FRET 0.8 and 0.6) and POST (FRET 0.2) states, which can be visualized when translocation is impaired. In the following, we denote the FRET 0.4 state as chimeric, CHI.

### Fluctuations between different states

**Figure 4:**
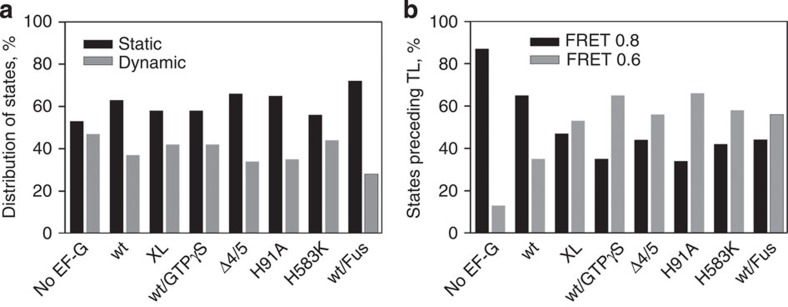
Conformational distribution of sub-populations immediately before the first FRET transition used for post synchronization. Distributions in PRE complexes were obtained without synchronization and are shown for comparison. (**a**) Static (black bars) and dynamic (grey bars) sub-populations. (**b**) FRET 0.8 (black bars) and 0.6 (grey bars) states before translocation (TL).

Next, we examined how EF-G engagement alters the dynamic properties of the ribosome in the PRE state by analysing the distribution between static (no transitions before photobleaching) and dynamic (fluctuating) ribosome sub-populations before and after the transition point used for post synchronization. Transitions towards the lower FRET values (0.4 or 0.2 states after addition of EF-G) were observed for both dynamic and static PRE complexes, indicating that both types of complexes were active in translocation (Fig. 4a). Thus, the two sub-populations reflect the variability of complexes rather than the presence of inactive complexes. Before the first transition event used for synchronization, the distribution of PRE complexes between static and dynamic sub-populations was the same for all EF-G variants (Fig. 4a). Furthermore, transitions to the lower FRET states occurred from either FRET 0.8 or FRET 0.6 states (Fig. 4b). This suggests that EF-G–GTP can bind to either C or H states and promote translocation, consistent with previous smFRET and ensemble kinetic results^23^,^34^,^44^,^46^,^47^. Compared with the PRE state without EF-G, in which the FRET 0.8 state was predominant, the addition of wt EF-G with GTPγS, or of EF-G mutants with GTP increased the relative population of the FRET 0.6 state. This reflects the stabilization of the H-state with FRET 0.6 by binding of EF-G^6^,^29^. The observation that the distribution between FRET 0.8 and 0.6 states was shifted—whereas translocation can start from either state—suggests that in the states before the first transition to the FRET 0.4 state EF-G was already bound, but the tRNAs had not yet moved to a CHI state. We thus distinguish between the initial (presumably readily reversible) EF-G binding to either C and H state, which we infer from the altered distribution of C and H states, and the following engagement of EF-G, which exerts a rearrangement of the complex coupled to tRNA movement.

**Figure 5:**
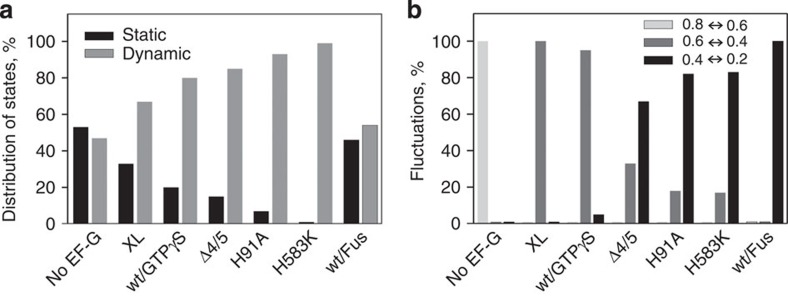
Conformational distribution of sub-populations after post synchronization. Distributions in PRE complexes were obtained without synchronization and are shown for comparison. (**a**) Distribution of static (black bars) and dynamic (grey bars) sub-populations. (**b**) Fluctuations between FRET 0.8↔0.6 states (light grey bars), FRET 0.6↔0.4 states (grey bars) and FRET 0.4↔0.2 states (black bars) within the dynamic sub-population.

After synchronization, that is, on complexes where EF-G was engaged in forward translocation, presumably by inducing the unlocking of the 30S subunit^28^, the portion of dynamic complexes increased dramatically (Fig. 5a and Table 1). This suggests that EF-G may promote translocation by rendering the ribosomes more dynamic. When translocation was slowed down by using EF-G mutants or replacing GTP with GTPγS, we observed fluctuations between FRET 0.4↔0.6 or 0.4↔0.2 (Fig. 5b). Transitions from FRET 0.4 or 0.6 to 0.8 were not observed, indicating that EF-G engagement renders the initial FRET 0.8 state inaccessible, in agreement with the observation that EF-G binding halts the fluctuations between C and H states^34^. This finding suggests that the properties of the FRET 0.6 state with EF-G engaged in translocation differ from the FRET 0.6 PRE state without EF-G, despite equal FRET values. We therefore suggest that the FRET 0.6 state with EF-G engaged with the ribosome represents an early translocation intermediate, referred to as a chimeric state CHI1. Which further transitions between the FRET 0.6, 0.4 and 0.2 states were the most frequent depended on the EF-G variant and the nucleotide (GTP or GTPγS) used (Fig. 5b and Table 1). In the presence of EF-G(XL)–GTP or EF-G(wt)–GTPγS, most of the transitions occurred between FRET 0.6 and 0.4. At these conditions, the complex is stalled in a PRE state in which the peptidyl-tRNA is Pmn-unreactive^23^ and translocation is slow on both 30S and 50S subunits^24^,^44^. Therefore, the observed FRET 0.4 state must be another relatively early translocation intermediate, which we refer to as CHI2 (Fig. 7). With EF-G(H583K) and EF-G(H91A), most of the transitions (∼80%) were observed between FRET 0.4 and 0.2 states; the remaining transitions were between FRET 0.4 and 0.6 states (Fig. 5b and Table 1). This implies that with these EF-G mutants, translocation was actually blocked at the FRET 0.2 state, allowing the ribosomes to reversibly sample the preceding FRET 0.4 and 0.6 states. Biochemical evidence indicates that complexes with EF-G(H583K) or EF-G(H91A) are blocked in an intermediate state, a translocation intermediate in which the peptidyl-tRNA 3′-end has moved towards the P-site into a state that is not yet Pmn reactive, whereas the tRNA anticodon remained in the A-site of the 30S subunit^15^,^23^. Thus, EF-G(H583K) and EF-G(H91A) stabilize the ribosome in a FRET 0.2 state, referred to as CHI3, which differs from the state (CHI2, FRET 0.4) stalled by EF-G(XL)–GTP or EF-G–GTPγS. In the presence of EF-G(Δ4/5), we observed FRET fluctuations between FRET 0.6 and 0.4 (32%), as well as between FRET 0.4 and 0.2 (65%), suggesting that the truncated factor stalls both CHI2 and CHI3 intermediates. Fus stabilized the tRNAs in POST positions with respect to both the 50S subunit and the body of the 30S subunit, but in a chimeric position with respect to the 30S subunit head^30^,^48^. In the presence of Fus, the complex fluctuated between FRET 0.2 and 0.4 states. In all cases, the transitions between the FRET 0.6, 0.4 and 0.2 states were rapid (Table 1 and Supplementary Fig. 3), suggesting a flat energy landscape between the EF-G-induced discrete states. Given the limited time resolution of TIRF experiments, these values probably represent lower limits for the transitions rates, but are overall consistent with translocation rates measured at room temperature in a similar buffer^23^.

### FRET between two tRNAs

**Figure 6:**
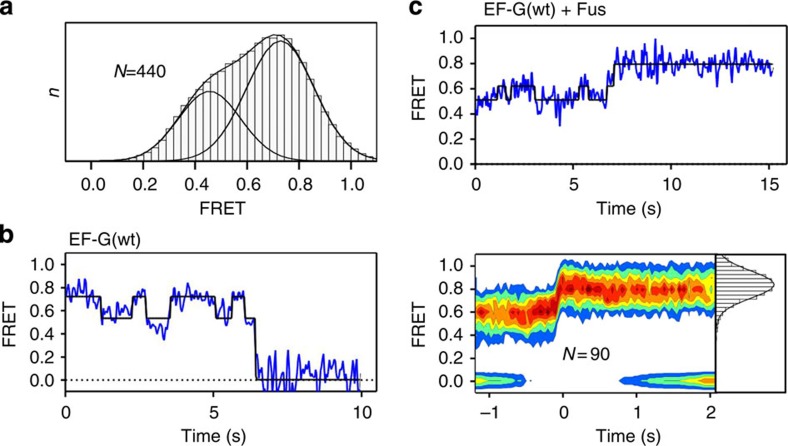
FRET between two tRNAs. (**a**) smFRET distribution histogram for the PRE complex with tRNA^fMet^(Cy3) in the P site and fMetPhe-tRNA^Phe^(Cy5) in the A site. The two populations had FRET efficiencies of 0.73±0.13 (FRET 0.7 state) and 0.46±0.12 (FRET 0.5 state). (**b**) Trajectory of smFRET on addition of EF-G. The transition to FRET 0 represents the dissociation of tRNA^fMet^(Cy3) from the E site in the course of translocation^5^. (**c**) The trajectory of smFRET on translocation with wt EF-G and Fus (top panel). Two-dimensional contour plot after post synchronization (bottom panel), showing the population with FRET efficiency of 0.85±0.12. *N* is the number of smFRET trajectories used to construct contour plot and FRET distribution histogram (grey bars). a.u., arbitrary units.

In the experiments with labels at the A-site tRNA and L11 (‘Lt-FRET'), the same FRET values were observed for the late CHI and POST states. In principle, these states can be differentiated based on structural, biochemical and rapid kinetic data^23^,^24^,^25^,^30^,^48^; for example, CHI3 and CHI4 states can be distinguished by the rates of the Pmn reaction of peptidyl-tRNA in the respective stalled complexes^23^. However, in the complex stabilized by Fus the 3′-end of the peptidyl-tRNA resides in the POST state, as it is readily reactive with Pmn^48^, which makes it difficult to distinguish between the CHI4 and POST states. To better discriminate between these states we used another established FRET pair^5^, this time with fluorescence labels on the tRNAs: tRNA^fMet^ labelled with Cy3 at thio-U8 and fMetPhe-tRNA^Phe^ with Cy5 attached to position 47 (‘tt-FRET'). As expected, PRE complexes in the absence of EF-G fluctuated between two states with tt-FRET efficiencies of 0.7 and 0.5, which were previously shown to represent PRE(C) and PRE(H) states of tRNAs, respectively^5^ (Fig. 6a). Rapid translocation induced by wt EF-G resulted in the loss of FRET due to dissociation of tRNA^fMet^ from the E site (Fig. 6b), consistent with previous data^5^. In contrast, addition of EF-G with Fus induced the formation of a stable tt-FRET 0.9 state (Fig. 6c). Thus, the CHI(POST) state stabilized by Fus and the true POST state are clearly distinct by their FRET values, 0.9 and 0, respectively. Unfortunately, tt-FRET between the two tRNA labels is not suitable to differentiate between CHI4 and other chimeric states, probably because the distance change is too small to be resolved.

## Discussion

**Figure 7:**
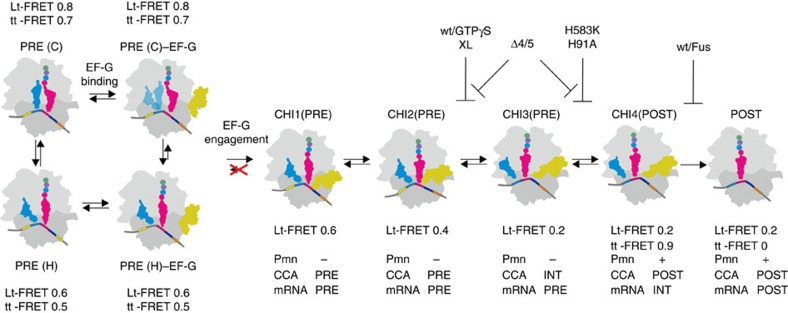
Intermediate states of tRNAs on ribosomes during EF-G-induced translocation. The 30S subunit is shown in grey, 50S subunit in light grey, P-site tRNA in blue, A-site peptidyl-tRNA in raspberry with the nascent chain indicated by circles. In the absence of EF-G, the ribosome fluctuates between Lt-FRET 0.8 and 0.6 states, which represent the PRE (C) and PRE (H) states. For the purpose of the present discussion focusing on the A-site tRNA, PRE1-4 states are grouped into C, because they differ only in the orientation of the P-site tRNA, P/P to P/E, but have a similar position of the A-site tRNA; H is considered identical to the PRE5 state, because the tRNAs are in P/E and A/P states^10^ (Fig. 1). Recruitment of EF-G (gold) shifts the equilibrium towards the PRE (H)–EF-G state (Fig. 4b). Engagement of EF-G blocks Lt-FRET 0.6→0.8 transitions (red cross). Intermediates CHI1 to CHI4 are identified based on the combination of FRET efficiencies and dynamic fluctuations (FRET; Table 1), the Pmn reactivity of peptidyl-tRNA (Pmn) and fluorescence changes of environmentally sensitive labels, that is, Bodipy attached to the N terminus of the nascent chain (CCA) and Alexa405 attached to the 3′-end of the mRNA (mRNA)^22^,^23^. CHI4 and POST states are distinguished based on the tt-FRET efficiency difference. The structure of the CHI4(POST) state stalled by Fus is known^30^. With EF-G(wt), transitions from CHI1(PRE) to CHI4(POST) are rapid and observed as a single step, whereas the transition from CHI4(POST) to POST can be distinguished as a separate kinetic step^22^,^23^,^69^.

Based on smFRET between labels attached to peptidyl-tRNA and to ribosomal protein L11 (Lt-FRET), we have identified several intermediate states of EF-G-induced tRNA translocation monitored from the perspective of the A-site peptidyl-tRNA moving in a step-wise manner through the ribosome from the PRE states (C or H) to the POST state (Fig. 7). The states can be distinguished based on (i) FRET efficiencies; (ii) the dynamic properties of the states, for example, their ability to fluctuate towards other states; and (iii) biochemical and kinetic properties of the stalled intermediates^15^,^22^,^23^. This way, the four states identified by different FRET efficiencies (0.8, 0.6, 0.4 and 0.2) fall into further sub-states, resulting in a total of nine different states, including three chimeric PRE states and one chimeric POST state (Fig. 7).

The binding of EF-G to the ribosome initially leads to the stabilization of the PRE(H) state, which alters the relative population of the PRE states (Figs 7, 4b and ref. 34). The fluctuations of the P/E-site tRNA and L1 are suppressed by EF-G binding as well^6^,^16^,^29^,^49^, suggesting an allosteric effect of EF-G engagement on the dynamics of the ribosome^50^. Concomitantly, GTP hydrolysis takes place^15^,^26^,^44^. At some point after the initial recruitment of EF-G, tRNAs start to move, which we observe as a change in Lt-FRET efficiency from 0.6/0.8 through 0.4 to 0.2 in the POST state. The intermediate FRET 0.4 state can be isolated when translocation is stalled at different states before completion. The intermediate can interconvert with the FRET 0.6 or FRET 0.2 states. Notably, the FRET 0.6 state sampled from FRET 0.4 does not fluctuate towards the high FRET 0.8 state, that is, the dynamic properties of this FRET 0.6 state are different from PRE(H), despite the same FRET efficiency. We suggest that at the onset of translocation, EF-G engages with the ribosome in a way that blocks backward fluctuations towards the C states. We thus refer to the FRET 0.6 state that does not interconvert with the FRET 0.8 state as a separate translocation intermediate, CHI1. The existence of two distinct binding states of EF-G, one resulting in only transient EF-G interaction with the ribosome and another leading to translocation, has been recently observed using smFRET labels on EF-G and ribosomal protein S12 (ref. 27). PRE(H)-EF-G or CHI1 may resemble the complex stalled in the presence of the antibiotic viomycin studied by cryo-EM^39^; we note that even if the FRET 0.4 state was sampled by the viomycin-stalled complex, this state was not captured by that cryo-EM reconstruction, which represented only a small fraction (<3%) of all complexes and was assigned by the authors as PRE5–EF-G state.

In the next transition, the tRNAs move to a CHI2 state with FRET 0.4, which is distinct from the other PRE states (C and H, FRET 0.8 and 0.6, and CHI1, FRET 0.6) and the POST (FRET 0.2) state. Transitions from CHI2 to the FRET 0.2 states are essentially abolished with EF-G(XL), indicating the formation of a stalled intermediate. This intermediate is not Pmn reactive^24^. Similarly, when the CHI2 state is stalled by the non-hydrolysable GTP analogue and the EF-G(Δ4/5) mutant, the Pmn reaction is very slow^23^,^45^ and there is practically no 50S translocation as reported by a label on peptidyl-tRNA^23^. Thus, the CHI2 and, by inference, CHI1 states are closer to PRE than to POST (Fig. 7).

In the next step, CHI2 rearranges to CHI3 with FRET 0.2; in that state, the peptidyl-tRNA remains in a PRE state according to the lack of Pmn reactivity of the intermediates stalled by EF-G(H91A) and (H583K) in the time-resolved Pmn assay^22^,^23^,^25^. In CHI3, the tRNA body has moved further away from protein L11 (Lt-FRET decreased from 0.4 to 0.2) and the 3′-end of the tRNA has moved, as suggested by the environmentally sensitive Bodipy probe, whereas the position of the mRNA relative to the 30S body has not changed appreciably^22^,^23^. Structurally, that or a similar state may correspond to the ribosome–EF-G complex trapped by neomycin to block completion of translocation and Fus to prevent dissociation of EF-G^31^. The exact placement of the neomycin-stalled intermediate on the translocation pathway is uncertain due to the presence of multiple binding sites for neomycin causing bimodal effects (inhibition at low and rescue at high concentrations) on the Pmn reactivity of peptidyl-tRNA^51^.

The movement of the peptidyl-tRNA into the chimeric POST state, CHI4 (Fig. 7), is not observed as an additional Lt-FRET change, but leads to a biochemically distinct complex with the peptidyl-tRNA positioned in the Pmn-reactive state in the P site. CHI4 is captured by the Fus-stalled structure visualized by cryo-EM^30^. Our results suggest that the two tRNAs in the P and E sites assume a unique arrangement in which their elbow regions are very close to each other (tt-FRET 0.9). The complex rearranges to the final POST state on release of EF-G and deacylated tRNA^52^, which leads to the disappearance of tt-FRET^5^.

The presence of the intermediate FRET 0.4 state in the Fus-stabilized complex is surprising, because extent and rate of translocation (as measured by the Pmn reaction, fluorescence changes of the A-site tRNA and toeprinting assays) are not affected by Fus^48^,^53^. However, it is consistent with a recent cryo-EM structure, which shows that Fus stalls the ribosome in a CHI state^30^. One potential explanation is that the transition from CHI3 to CHI4 is intrinsically rapid (see below) and thus the observed rate of the Pmn reaction or the fluorescence change is determined by the preceding steps, which are not affected by Fus. This interpretation is also consistent with the notion that conformational changes that precede the actual tRNA movement (‘unlocking') are rate limiting for translocation^28^, which would also explain why only one dominant decay time was found in the dwell time analysis.

When EF-G–GTP is added to the PRE complex, none of the chimeric intermediate states is long-lived enough to be detectable, as translocation is rapid and driven towards POST on both subunits synchronously^23^. The intermediate states become detectable when the coupling between the conformational changes of EF-G and the ribosome is disrupted. Restoring the coupling, for example, by reducing the disulfide bond in EF-G(XL), allows for rapid transition of the stalled CHI2 intermediate towards the POST state, suggesting that CHI1 and CHI2 states are on the pathway. The same is probably true for the CHI3 state, because EF-G mutations that stabilize the CHI3 intermediate slow down translocation only moderately (30-fold), rather than blocking it completely^22^,^25^.

EF-G contributes to tRNA translocation in several ways. Initial binding of EF-G to PRE complexes favours the H state, consistent with the reported stabilization of the H state by EF-G binding^6^,^16^,^29^. The ribosomes in both C and H states can bind EF-G and proceed towards translocation, which is in apparent disagreement with the notion that only the H state was capable of EF-G binding^43^,^54^, but in line with other smFRET and ensemble kinetic work^23^,^34^,^46^. We assume that the state of the ribosome immediately after initial EF-G binding may have escaped detection due to limited time resolution; rather, a later state may be observed representing an intermediate where EF-G is already engaged. In support of this notion, the times of EF-G arrival to rotated/hybrid and non-rotated/classical ribosomes were similar in the presence of a non-hydrolysable GTP analogue^43^, possibly indicating a true pre-hydrolysis EF-G recruitment step. In contrast, the following engagement step appears to abolish any dynamic fluctuations of the H state back to the C state, suggesting that the dynamic properties of the complex are dramatically altered, despite the unchanged FRET.

The present results suggest how the conformational rearrangements of EF-G are coupled to the stepwise movement of the tRNAs through the ribosome. Precluding fluctuations to the C state may be important for providing the directionality of translocation, which in the absence of the factor is inherently reversible and governed by the thermodynamic gradient of tRNA binding to the A, P and E sites^10^,^18^,^55^,^56^,^57^,^58^. In fact, translocation can be dramatically accelerated by promoting directionality, for example, by making reverse transitions unfavourable^57^. In blocking the backward transition to the C state, EF-G acts similar to the pawl in a Brownian machine. EF-G engagement also favours the transition to CHI2, which thermodynamically is a downhill process (Fig. 7 and Table 1). The population distribution—expressed in terms of *k*_B_*T*—suggest that fluctuations between CHI1, CHI2, CHI3 and CHI4 states are accessible within the 2-*k*_B_*T* limit indicative of thermally driven fluctuations, unless the respective downstream step is blocked (Fig. 7). The flat translocation landscape that connects all CHI states requires GTP hydrolysis, conformational rearrangements of EF-G that couple GTP hydrolysis to the movement of domain 4 and rearrangements of the 30S subunit induced by interactions with the tip of domain 4, in agreement with structural and kinetic data^19^,^20^,^21^,^24^,^25^,^44^. As GTPγS and EF-G(H91A) stall translocation in different CHI states, although in both cases there is no GTP hydrolysis, it appears that not only GTP hydrolysis *per se* but also the details of the mechanics of conformational coupling are important. EF-G binding also converts PRE complexes that were static, that is, not sampling between C and H states for several seconds, into dynamic ones, which rapidly fluctuated between various chimeric states. Thus, the factor acts as an energizer of ribosome motions. The intermediate states identified in this work represent snapshots that illustrate how the energy of EF-G–GTP binding and GTP hydrolysis is coupled to stepwise movements of the tRNAs through the ribosome.

## Methods

### Materials

Initiation factors (IF1, IF2 and IF3), elongation factors (EF-Tu and EF-G) and tRNAs were from *Escherichia coli* and were prepared as described^23^,^59^,^60^. Biotin-labelled mRNA was purchased from Thermo Scientific (5′-Biotin-CAACCUAAAACUUACACACCCGGUAAGGAAAUAAAA AUG UUU AAA CGU AAA UCU ACU-3′).

### Labelling of L11

For the expression and purification of recombinant protein L11, a published protocol^61^ was modified as follows. Protein L11 was expressed in *E. coli* BL21(DE3) after induction with isopropyl-β-D-thiogalactoside (1 mM). Cells were lysed by sonication in buffer (50 mM HEPES, 10 mM MgCl_2_, 10 mM NH_4_Cl, 1 mM dithiothreitol, 0.5 mM EDTA, pH 7.2) and inclusion bodies containing L11 were solubilized by dissolving the cell pellet in the same buffer containing additionally 6 M urea. The protein was dialysed against 100 volumes of the same buffer and purified by fast protein liquid chromatography using a HiTrap SP HP column (GE Healthcare) using a linear gradient of 10–500 mM NH_4_Cl in buffer as above, with 6 M urea. Cy3-labelling of L11 at the single native cysteine at position C38 was carried out in the same buffer with 6 M urea and 0.5 M NH_4_Cl by adding a threefold excess of Cy3-maleimide (GE Healthcare) dissolved in dimethylsulfoxide and incubating for 12 h at 4 °C. Excess dye was removed on a HiTrap SP HP column using the same salt gradient as above. To refold labelled L11, the protein was rebuffered by centrifugation into 50 mM HEPES, 10 mM MgCl_2_, 300 mM NH_4_Cl, 1 mM dithiothreitol, 0.5 mM EDTA, pH 7.2, 25% glycerol using a Vivaspin 5,000 concentrator. The fluorescence anisotropy of Cy3 was measured in a Fluorolog-3 fluorimeter (Horiba) equipped with polarizers for both excitation and emission light paths. The anisotropy value of 0.300±0.005, measured for Cy3-labelled ribosomes without or with EF-G bound was close to the value of 0.386 that was reported for the limiting anisotropy of Cy3 (ref. 62).

### Ribosome preparation

Ribosomes lacking protein L11 (70S(ΔL11)) were purified from *E. coli* strain AM68 (ref. 63) according to a standard protocol^64^. 70S(ΔL11) were reconstituted by incubation with a fivefold excess of fluorophore-labelled L11 in buffer (50 mM Tris-HCl, 70 mM NH_4_Cl, 30 mM KCl, 20 mM MgCl_2_, pH 7.5) for 45 min at 45 °C. Excess L11 was removed by centrifugation through a 1.1-M sucrose cushion in the same buffer. Pellets of reconstituted 70S ribosomes were dissolved and stored in the same buffer, but containing 7 mM MgCl_2_.

### Labelling and purification of Phe-tRNA^Phe^(acp47-Cy5)

Cy5-labelling at the aminocarboxypropyl group at residue 47 was carried out by incubating tRNA^Phe^ from *E. coli* with a 100-fold excess of Cy5-succinimidylester (GE Healthcare) in 20 mM HEPES, pH 8, for 12 h at 37 °C. Excess dye was removed by phenol extraction and ethanol precipitation. tRNA^Phe^(acp47-Cy5) was aminoacylated and purified by HPLC^59^.

### Sample preparation

Initiation complex formation was carried out by incubating 0.1 μM ribosomes with a 1.7-fold excess of IF1, IF2 and IF3, a 3-fold excess of fMet-tRNA^fMet^ and mRNA, and 1 mM GTP in buffer (50 mM Tris-HCl, 70 mM NH_4_Cl, 30 mM KCl, 7 mM MgCl_2_, pH 7.5) for 30 min at 37 °C. To prepare the ternary complex, 0.2 μM EF-Tu was incubated with 1 mM GTP, 3 mM phosphoenolpyruvate and 0.1 mg ml^−1^ pyruvate kinase for 15 min at 37 °C, followed by the addition of 0.1 μM Phe-tRNA^Phe^(acp47-Cy5). PRE complexes were formed by mixing initiation and ternary complexes and incubating for 1 min at room temperature. If not stated otherwise, translocation was initiated by the addition of EF-G or EF-G mutant (to 0.1 μM concentration) and 1 mM GTP. Fus was used at a concentration of 0.2 mM.

### Cover slip preparation

Cover slips and objective slides were cleaned by bath sonication in 1 M KOH and exposure to plasma (FEMTO plasma cleaner, Diener Electronic GmbH, Germany). Surfaces were then silanized by sonication in 3.9 mM N1-[3-(trimethoxysilyl) propyl] diethylenetriamine (Sigma-Aldrich) and 1.7 mM acetic acid, and baked for 20 min at 120 °C. PEG/PEG-Biotin functionalization of silanized surfaces was carried out by incubation with 20 mM PEG-NHS (MeO-PEG-NHS, IRIS Biotech GmbH, PEG1165), 0.2 mM Biotin-PEG-NHS (IRIS Biotech, PEG1057) and 20 mM KOH in 100 mM H_3_BO_3_ solution for 1 h at room temperature. Excess PEG was removed by 1 min sonication in H_2_O. Cover slips were dried at 60 °C and stored under vacuum. For TIRF experiments, flow chambers were generated by combining objective slides and cover slips with double-sided sticky tape.

### Sample preparation for TIRF

Ribosome complexes were diluted to 1 nM with TIRF buffer (50 mM Tris-HCl, pH 7.5, 70 mM NH_4_Cl, 30 mM KCl, 15 mM MgCl_2_, 1 mM spermidine and 8 mM putrescine). Biotin/PEG-functionalized cover slips were incubated for 5 min at room temperature with the same buffer containing additionally 10 mg ml^−1^ BSA and 1 μM neutravidin (Thermo Scientific). Excess neutravidin was removed by washing the cover slip with the same buffer containing 1 mg ml^−1^ BSA. Ribosome complexes were applied to the surface and immobilized through the mRNA–biotin–neutravidin interaction. Images were recorded after the addition of imaging buffer to the sample. FRET signals reporting on the time course of translocation were obtained by adding 0.1 μM EF-G and 1 mM GTP to the imaging buffer (TIRF buffer with 2.5 mM protocatechuic acid, 50 nM protocatechuate-3,4-dioxygenase (from *Pseudomonas*), 1 mM trolox (6-hydroxy-2,5,7,8-tetramethylchromane-2-carboxylic acid) and 1 mM methylviologen (Sigma-Aldrich)). A fivefold excess volume of the solution containing EF-G was sufficient to rapidly manually exchange the volume of the flow chamber (about 10 μl) before starting the imaging and was optimized to obtain a maximum number of translocation events.

### TIRF microscopy

TIRF imaging was performed on an IX 81 inverted microscope using a PLAPON 60 × 1.45 numerical aperture objective (Olympus, Japan). Fluorescence was excited by a 561 nm solid-state laser operated at a power of 25 mW. Images were recorded with an electrom multiplying CCD (charge-coupled device) camera (CCD-C9100-13, Hamamatsu, Japan). In FRET experiments, colour channels were separated by projecting donor and acceptor emission on different parts of the CCD chip using an image splitter (dual view micro imager DV2, Photometrics, USA), filter specifications HQ 605/40, HQ 680/30 (Chroma Technology). If not stated otherwise, movies were recorded at a rate of 30 frames per second. The experiments were carried out at 22 °C.

### Data analysis

Fluorescence time courses for donor (Cy3) and acceptor (Cy5) were extracted using custom-made Matlab (MathWorks) software according to published protocols^16^,^37^. A semi-automated algorithm (Matlab) was used to select anti-correlated fluorescence traces exhibiting characteristic single fluorophore intensities. The bleed-through of Cy3 signal into the Cy5 channel was corrected using an experimentally determined coefficient (∼0.13 in our setup). All trajectories were smoothed over three data points and truncated to remove photobleaching and photoblinking events. Traces with lifetimes of Cy3 or Cy5 less than ten frames (0.33 s) or with multiple photobleaching steps were excluded from the analysis. The FRET efficiency was defined as the ratio of the measured emission intensities, Cy5/(Cy3+Cy5)^5^,^7^,^37^. Trajectories of smFRET were fitted by Hidden Markov model using the vbFRET software package (http://vbfret.sourceforge.net/)^65^, which finds the most probable fit as well as the number of states by applying an expectation-maximization algorithm and a variational Bayesian analysis, thereby avoiding overfitting of data^65^,^66^. Models with different number of states (*K*=1, …, *n*+1, where *n* is the expected number of states) were considered for each data set. FRET changes of <0.05 in idealized trajectories were not considered as transitions, as such changes were smaller than the s.d. of the Gaussian distributions and usually were not distinguished from the noise. Transitions lasting for only one frame were not included in the analysis as well. About 3% of all traces were poorly idealized by hidden Markov modelling (probably due to insufficient length of traces or noise) and eliminated from subsequent analysis.

Two-dimensional contour plots were generated from time-resolved FRET trajectories. The set of all FRET traces for a given complex was compiled in a histogram, which was fitted to a sum of Gaussian functions. Matlab code using an unconstrained nonlinear minimization procedure (fminsearch, Matlab, R2011b) yields mean values and s.d. for the distribution of FRET states. For the experiments with EF-G FRET, traces were post synchronized relative to the first transition to FRET≤0.5. Histograms were constructed for the first 45 frames (1.5 s) after post synchronization.

Dwell times of different FRET states of fluctuating traces were extracted from idealized trajectories^42^. The dwell time histogram for each transition was fitted to an exponential function, *y*=*y*_0_+*Ae*^−*t*/*τ*^. Rates (*k*) were calculated by taking the inverse of dwell times (*τ*). The mean dwell times (<0.5 s) for all data sets were considerably smaller than the observed mean length of FRET traces until photobleaching took place (mean trace length ∼10 s). Correction of transition rates considering the static traces and the rates of photobleaching was carried out as described^67^,^68^:


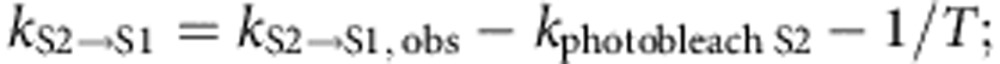



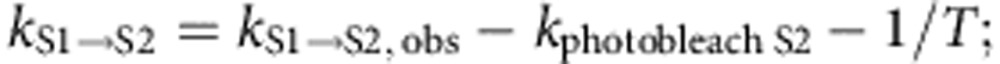


where *k*_S2_→_S1_ and *k*_S1→S2_ are corrected transition rates values, *k*_S2_→_S1, obs_ and *k*_S1_→_S2, obs_ are transition rates calculated from the dwell time histograms, *k*_photobleach S1_ and *k*_photobleach S2_ are the photobleaching rates of states S1 and S2, respectively, and *T*=33 s (observation time). The difference between *k*_obs_ and the corrected *k* values was relative small.

### Structure modelling

The distances between the fluorophores in different states were estimated from atomic models of translocation intermediates obtained by cryo-EM and X-ray crystallography^10^,^30^,^39^,^40^ by measuring the distance between Cα of Cys38 of protein L11 and C1' of U47 of the peptidyl tRNA. To model the position of L11 in cryo-EM structures PRE1a, PRE4, PRE5b and POST4 (PDB 3j4v/3j52, 3j53/3j54, 3j57/3j58 and 3j5j/3j5k, respectively^10^,^18^), which show only scattered densities in that region, the position of L11 was taken from the best-resolved reconstruction of the factor-free cryo-EM map of *E. coli* PRE complex stabilized by Vio (PRE4-Vio, PDB 3j5u; ref. 39). The atomic models for PRE1, PRE4, PRE5b and POST4 were aligned through the 50S subunit onto the structure of the PRE4-Vio complex using the density fit option in UCSF Chimera software with models filtered to 5 Å resolution. Protein L11 and its ribosome binding region (residues 1,048 to 1,103 of 23S rRNA) were replaced by the aligned coordinates of the PRE4-Vio complex. The complexes with EF-G stalled by Fus (PDB 3j5n/3j5o^30^ and 2wri/2wrj 40) are shown for comparison (Fig. 1).

## Additional information

**How to cite this article:** Adio, S. *et al*. Fluctuations between multiple EF-G-induced chimeric tRNA states during translocation on the ribosome. *Nat. Commun.* 6:7442 doi: 10.1038/ncomms8442 (2015).

## Supplementary Material

Supplementary Information Supplementary Figures 1-3, Supplementary Tables 1-2 and Supplementary References

## Figures and Tables

**Table 1:** Distribution and transition rates of intermediate states during translocation*.

EF-G	*P*_0.6_/*P*_0.4_†	*K*_eq 0.4/0.6_‡	*k*, s^−1^ (*n*)§	*P*_0.4_/*P*_0.2_	*K*_eq 0.2/0.4_‡	*k*||, s^−1^ (*n*)	*k*||, s^−1^ (*n*)	*k*_off_, s^−1^
			0.6→0.4			0.4→0.2	0.6→0.2	For state
			0.4→0.6			0.2→0.4	0.2→0.6	FRET 0.4
XL	0.13/0.87	6.7	7.9±0.2 (840)	—	—	—	–	–
			*1.2*¶ (820)					
wt/GTPγS	0.06/0.92	15.3	6.9±0.2 (728)	0.92/0.02	0.02	0.8±0.4 (42)	—	≤0.07#
			*0.5*¶ (745)			4.2±1.6 (37)		
Δ4/5	0.08/0.67	8.4	6.0±0.2 (218)	0.67/0.25	0.37	2.9±0.4 (471)	3.7±2.5 (28)	≤0.08#
			3.0±0.1 (226)			7.6±0.2 (439)	6.2±5.0 (16)	
H91A	0.06/0.67	11.2	7.2±0.4 (81)	0.67/0.27	0.40	3.9±0.2 (402)	4.8±3.7 (28)	1.0
			4.2±0.8 (79)			7.2±0.5 (345)	7.0±4.1 (21)	
H583K	0.12/0.56	4.7	7.1±0.6 (63)	0.56/0.32	0.57	4.7±0.1 (347)	3.2±1.9 (29)	0.9
			4.7±0.2 (65)			7.9±0.3 (274)	5.9±4.1 (18)	
wt/Fus	—	—	—	0.64/0.36	0.56	3.8±0.2 (493)	–	–
						4.3±0.2 (440)		

^*^All values were calculated after post synchronization of FRET time courses.

^†^*P*, relative populations of states.

^‡^*K*_eq_=*P*_S2_/*P*_S1_.

^§^*n*, number of transitions.

^||^Mean value±s.d.

^¶^In cases indicated by italics, *k*_0.4→0.6_ was determined from the respective *K*_eq 0.4/0.6_ and *k*_0.6→0.4_.

^#^Rate was obtained in experiments with integration times ≥100 ms.
