# Supplementary material for: Fluctuations between multiple EF-G-induced chimeric tRNA states during translocation on the ribosome
Source: Nat Commun. 2015 Jun 15;6:7442. doi: 10.1038/ncomms8442 (PMC4490557; doi:10.1038/ncomms8442)
Supplement: Supplementary Information — Supplementary Figures 1-3, Supplementary Tables 1-2 and Supplementary References [file ncomms8442-s1.pdf]

Supplementary Data for the Manuscript:

Fluctuations between multiple EF-G-induced chimeric tRNA states during translocation on the ribosome

Sarah Adio[1†], Tamara Senyushkina[1†], Frank Peske[1], Niels Fischer[2], Wolfgang Wintermeyer[1*] and Marina V. Rodnina[1*]

[1]Department of Physical Biochemistry and [2]3D Electron Cryomicroscopy Group, Max Planck Institute for Biophysical Chemistry, 37077 Goettingen, Germany

Supplementary Figures

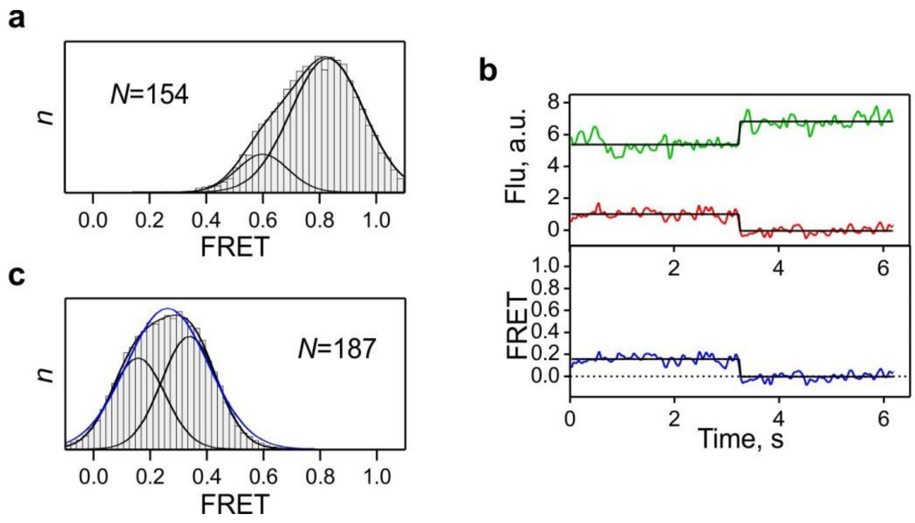

Supplementary Fig. 1 | Examples of smFRET distribution histograms and time traces.

(**a**) smFRET distribution histogram for the PRE complex measured at 130 ms time resolution.

(**b**) Example of single-molecule fluorescence intensity trajectories in the POST complex. Cy3 (green) and Cy5 (red) (top panel) and the trajectory of smFRET (bottom panel) showing photobleaching of the acceptor.

(**c**) smFRET distribution histogram of the Fus-stabilized complex evaluated with a sum of two Gaussian terms (black lines) compared to a single-Gaussian fit (blue line). *n* is the number of events.

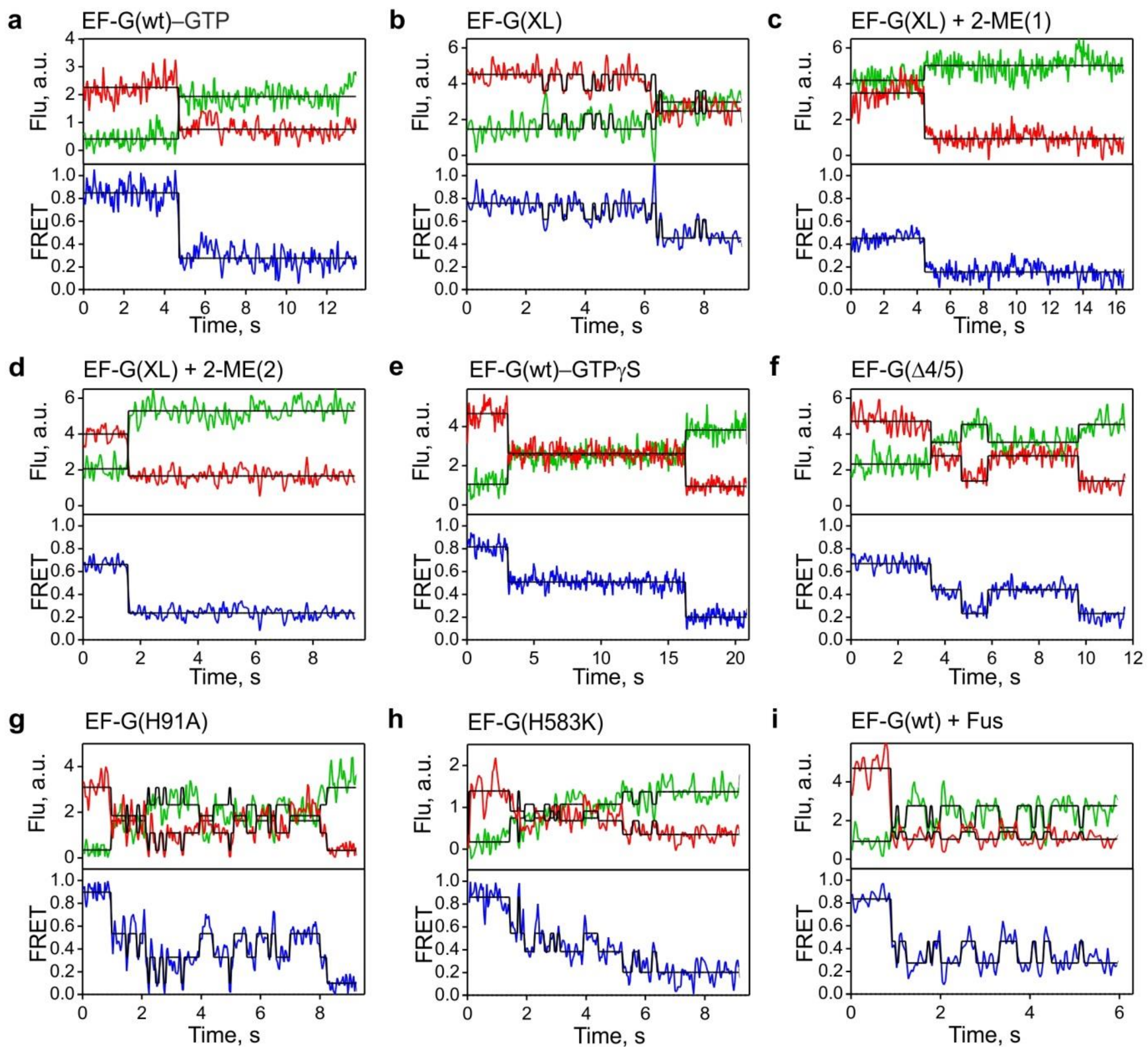

Supplementary Fig. 2 | smFRET in the presence of EF-G.

Representative examples of single-molecule fluorescence intensity (Flu) trajectories for Cy3 (green) and Cy5 (red) and the trajectory of smFRET (blue) evolution over time. (**a-i**) in the presence of wt or mutant EF-G, GTP or GTPγS, or after addition of Fus, as indicated. (**c**) and (**d**) show two different translocation scenarios after re-activation of EF-G(XL) by treatment with 2-mercaptoethanol (2-ME) when the disulfide bridge was reduced in EF-G bound to the complex with tRNAs in FRET 0.4 state (**c**) or in free EF-G (**d**). a.u., arbitrary units.

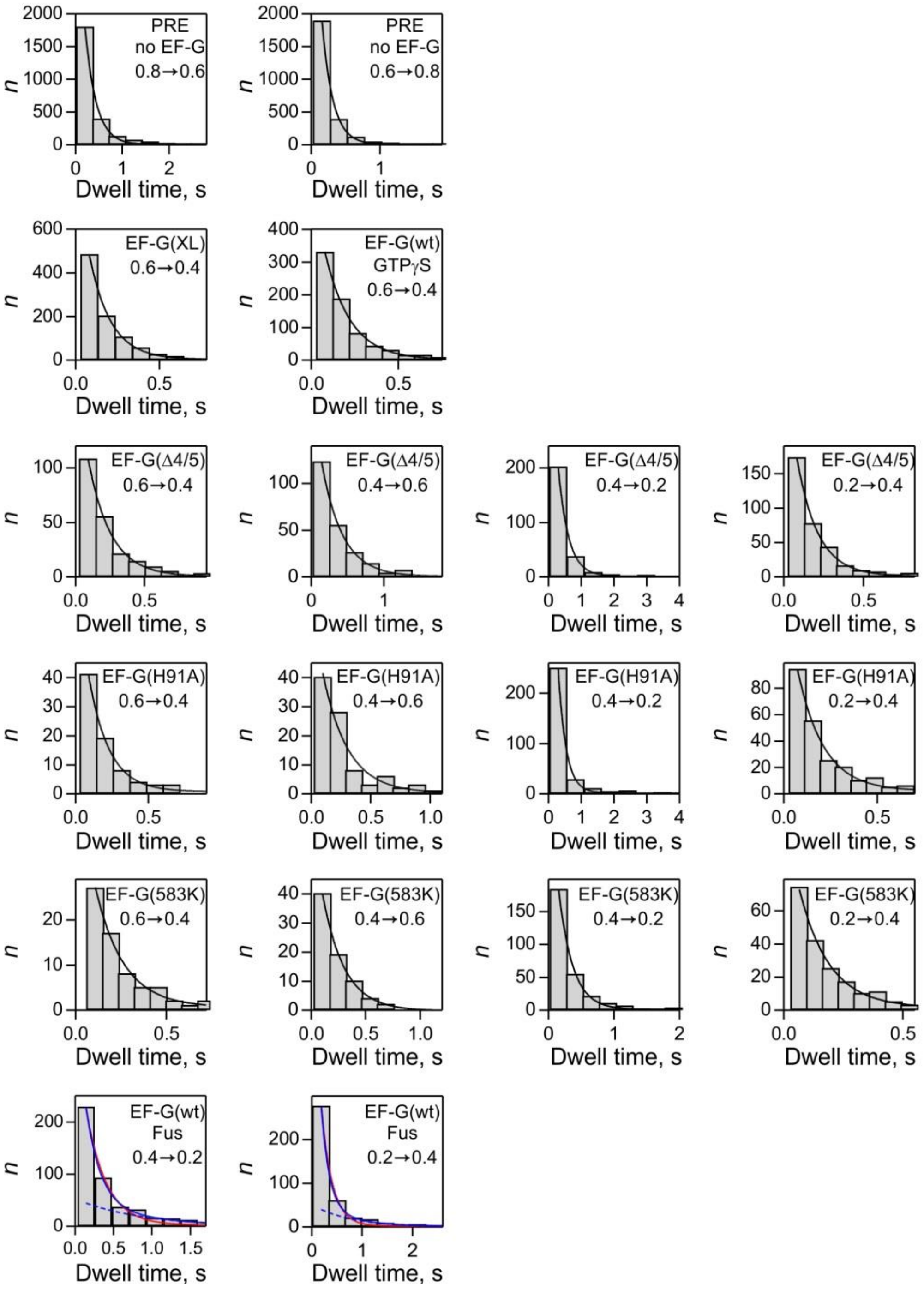

Supplementary Fig. S3 | Dwell time analysis of complexes as indicated.

The bin size was optimized to reduce chi squared[1]. For the Fus-stalled complex, results of one-exponential (red lines, R^2=0.9921 for 0.4→0.2 and R^2=0.9969 for 0.2→0.4) and two-exponential

(blue) fitting are compared. Parameters of two-exponential fits: 0.4→0.2 FRET, k_1 = 5.4 ± 0.5 s^{-1}, k_2 = 1.2 ± 0.3 s^{-1}, R^2= 0.9979; 0.2→0.4 FRET, k_1 = 6.2 ± 0.4 s^{-1}, k_2 = 1.3 ± 0.3 s^{-1}, R^2= 0.9995. Rates obtained by single-exponential fitting are summarized in Table 1. $k_{photobleach}$ was 0.31 s^{-1} for EF-G(Δ4/5), 0.32 s^{-1} for EF-G(H91A), 0.18 s^{-1} for the FRET 0.4 state with wt EF-G and Fus and 0.22 for the FRET 0.2 state with wt EF-G and Fus. $k_{photobleach}$ values for the PRE complex are in Supplemental Table 2.

Supplementary Tables

Supplementary Table 1 | Summary of EF-G mutants and inhibitors used in this work.

EF-G	Inhibitor	GTP hydrolysis	TL of tRNA CCA end	Pmn reaction	TL of mRNA	Reference
wt	–	Rapid	Rapid	Rapid	Rapid	[2-8]
XL	–	Rapid	n.d.	Very slow	Very slow	[9]
wt	GTPγS	Very slow	n.d.	Slow	Slow	[4,10]
Δ4/5	–	Rapid	Very slow	Very slow	Very slow	[4,11]
H91A	–	None	INT	Slow	Slow	[2,3]
H583K	–	Rapid	INT	Slow	Slow	[3,11]
wt	Fus	Rapid	n.d.	Rapid	Rapid to CHI	[4,12-14]

Supplementary Table 2 | Population distribution and transition rates for PRE sub-states in the absence of EF-G.

FRET pair (Mg^{2+})	All trajectories			Static			Dynamic			
	Dyn/Stat (N)[a]	P_C/P_H[b]	K_{eq}[c]	P_C/P_H[b] (N)	K_{eq}[c]	$k_{photobleach}$, s^{-1}, C/H	P_C/P_H[b] (N)	K_{eq}[c] C→H	k, s^{-1} C→H	k, s^{-1} H→C
Lt-FRET (15 mM)	0.47/0.53 (465)	0.87/0.13	0.15	0.96/0.04 (246)	0.04	0.15/0.27	0.66/0.34 (219)	0.52	4.1 ± 0.1	6.0 ± 0.1
Lt-FRET (7 mM)	0.57/0.43 (401)	0.57/0.43	0.75	0.73/0.27 (174)	0.37	0.13/0.13	0.53/0.47 (227)	0.89	4.5 ± 0.1	5.0 ± 0.1
tt-FRET (15 mM)	0.60/0.40 (440)	0.65/0.35	0.54	0.72/0.28 (174)	0.39	0.22/0.21	0.71/0.29 (266)	0.41	3.4 ± 0.1	4.3 ± 0.1

[a] N, number of trajectories. [b] P, relative populations of states; $P_C+P_H = 1$. [c] $K_{eq} = P_H/P_C$. $k_{photobleach}$, the photobleaching rates of states. C – classical state. H – hybrid state.

Supplementary References

1. Cornish, P.V., Ermolenko, D.N., Noller, H.F. & Ha, T. Spontaneous intersubunit rotation in single ribosomes. *Mol Cell* **30**, 578-88 (2008).
2. Cunha, C.E. et al. Dual use of GTP hydrolysis by elongation factor G on the ribosome. *Translation* **1**, e24315 (2013).
3. Holtkamp, W. et al. GTP hydrolysis by EF-G synchronizes tRNA movement on small and large ribosomal subunits. *EMBO J.*, Mar 10. [Epub ahead of print] (2014).
4. Rodnina, M.V., Savelsbergh, A., Katunin, V.I. & Wintermeyer, W. Hydrolysis of GTP by elongation factor G drives tRNA movement on the ribosome. *Nature* **385**, 37-41 (1997).
5. Savelsbergh, A. et al. An elongation factor G-induced ribosome rearrangement precedes tRNA-mRNA translocation. *Mol Cell* **11**, 1517-1523 (2003).
6. Khade, P.K. & Joseph, S. Messenger RNA interactions in the decoding center control the rate of translocation. *Nat Struct Mol Biol* **18**, 1300-2 (2011).
7. Pan, D., Kirillov, S.V. & Cooperman, B.S. Kinetically competent intermediates in the translocation step of protein synthesis. *Mol Cell* **25**, 519-29 (2007).
8. Liu, Q. & Fredrick, K. Contribution of intersubunit bridges to the energy barrier of ribosomal translocation. *Nucl Acids Res* **41**, 565-574 (2012).
9. Peske, F., Matassova, N.B., Savelsbergh, A., Rodnina, M.V. & Wintermeyer, W. Conformationally restricted elongation factor G retains GTPase activity but is inactive in translocation on the ribosome. *Mol Cell* **6**, 501-505 (2000).
10. Katunin, V.I., Savelsbergh, A., Rodnina, M.V. & Wintermeyer, W. Coupling of GTP hydrolysis by elongation factor G to translocation and factor recycling on the ribosome. *Biochemistry* **41**, 12806-12812 (2002).

11. Savelsbergh, A., Matassova, N.B., Rodnina, M.V. & Wintermeyer, W. Role of domains 4 and 5 in elongation factor G functions on the ribosome. *J Mol Biol* **300**, 951-961 (2000).

12. Peske, F., Savelsbergh, A., Katunin, V.I., Rodnina, M.V. & Wintermeyer, W. Conformational changes of the small ribosomal subunit during elongation factor G-dependent tRNA-mRNA translocation. *J Mol Biol* **343**, 1183-94 (2004).

13. Ermolenko, D.N. et al. The antibiotic viomycin traps the ribosome in an intermediate state of translocation. *Nat Struct Mol Biol* **14**, 493-7 (2007).

14. Stanley, R.E., Blaha, G., Grodzicki, R.L., Strickler, M.D. & Steitz, T.A. The structures of the anti-tuberculosis antibiotics viomycin and capreomycin bound to the 70S ribosome. *Nat Struct Mol Biol* **17**, 289-93 (2010).
